# Diagnostic Value of ^99m^Tc-MIBI Myocardial Perfusion Imaging in Detecting Myocardial Ischemia of Children with Kawasaki Disease and Coronary Artery Lesions

**DOI:** 10.1007/s00246-024-03545-2

**Published:** 2024-06-28

**Authors:** Yiting Gui, Yixiang Lin, Ha Wu, Guangan Dai, Xuecun Liang, Chen Chu, Yuanzheng Zheng, Quming Zhao, Feng Wang, Shuna Sun, Guoying Huang, Weili Yan, Lan He, Fang Liu

**Affiliations:** 1 Heart Center, Children’s Hospital of Fudan University, Shanghai, China; 2 Nuclear Medicine Department, Children’s Hospital of Fudan University, Shanghai, China; 3 Department of Clinical Epidemiology and Clinical Trial Unit, Children’s Hospital of Fudan University, Shanghai, China

**Keywords:** Kawasaki disease, Coronary artery lesions, Myocardial ischemia, Myocardial perfusion imaging, Children

## Abstract

Pediatric patients with coronary artery lesions (CALs) after Kawasaki disease (KD) may be complicated with myocardial ischemia. Although previous studies in adults have proven the diagnostic value of ^99m^Tc-MIBI myocardial perfusion imaging (MPI) for ischemic heart disease, its feasibility and accuracy in this pediatric population remain uncertain. In this retrospective study, we collected data of 177 pediatric patients (Age range: 6 months to 14 years) who had undergone MPI and coronary artery angiography (CAG) between July 2019 and February 2023. Using the positive result of CAG as the reference standard of myocardial ischemia, we compared the results of ^99m^Tc-MIBI MPI with other non-invasive examinations, including cardiac magnetic resonance imaging (CMRI), echocardiogram, and comprehensive electrocardiogram-related examinations. All patients finished adenosine triphosphate stress MPI without major side effects. The sensitivity of MPI was 79.17%, which was greater than CMRI and echocardiogram (*P* < 0.05). The negative predictive value and the accuracy of MPI were 89.9% and 71.75%, indicating the advantages over others. Composite monitoring strategy of MPI and CMRI effectively improved the diagnostic performance (*P* < 0.001). In 4 cases diagnosed with myocardial ischemia by “MPI + CMRI,” despite the absence of significant stenosis, multiple giant coronary artery aneurysms (GCAA) were all observed in CAG. ^99m^Tc-MIBI MPI is the preferred non-invasive examination for detecting myocardial ischemia in pediatric patients with CAL after KD. When combined with CMRI, it can enhance diagnostic accuracy. Multiple GCAAs without stenosis may be an isolated risk factor of myocardial ischemia.

## Introduction

Kawasaki disease (KD), also known as mucocutaneous lymph node syndrome, is a systematic vasculitis in children [1]. One of the most severe complications is coronary artery lesions (CALs), typically presenting as coronary artery dilatation and aneurysm formation [2]. From 2013 to 2017, the incidence of CALs following KD was 9.1%, with 2.7% for medium coronary artery aneurysms and 0.7% for giant coronary artery aneurysms (GCAA) in Shanghai, China [3]. CALs, especially GCAA, are prone to thrombosis formation, coronary artery stenosis, and occlusion, which can lead to myocardial ischemia, myocardial infarction, or even death [1]. Therefore, the systematic evaluation of myocardial perfusion and early diagnosis and treatment of myocardial ischemia are crucial for long-term management of pediatric patients with CALs after KD [1, 4, 5].

Selective coronary artery angiography (CAG) is the gold standard of CALs diagnosis [6, 7]. It allows for the evaluation of the degree of coronary artery anatomical stenosis, and indirectly reflects the blood flow to the corresponding myocardium. Fractional flow reserve (FFR) is effective for detecting functional myocardial ischemia and guiding coronary artery revascularization [6, 7]. However, it is an invasive and radioactive diagnostic technique that requires customized pressure guide wire and guiding catheter, making its use in young children extremely limited. Moreover, in cases of pediatric patients with GCAA after KD, due to the considerable difficulties of guide wire to pass through giant aneurysm as well as the risk of thrombus detachment, it is more challenging to perform FFR examination.

Myocardial perfusion imaging (MPI) [8] with single-photon emission computed tomography (SPECT) is a non-invasive imaging technique that visualizes local blood flow in the myocardium and predicts the presence of myocardial ischemia [9, 10]. In adult patients, recent studies have shown that the diagnostic sensitivity and specificity of ^99m^Tc-methoxyisobutylisocyanine (^99m^Tc-MIBI) MPI in detecting myocardial ischemia were reported to be 68–74% and 71–79%, respectively, in comparison with FFR (≤ 0.75) [11, 12]. Meanwhile, the diagnostic sensitivity and specificity of ^99m^Tc-MIBI MPI in detecting myocardial ischemia may reach 73–96% and 70–89%, respectively, in adult patients with coronary artery stenosis (≥ 50%) confirmed by CAG [13]. In pediatric patients, despite there are studies on the application of ^99m^Tc-MIBI MPI for detecting myocardial ischemia [14, 15], there is a lack of systematic evaluation of ^99m^Tc-MIBI MPI and comparison with other diagnostic methods in such patients.

By incorporating CAG results, we analyzed and compared the results of ^99m^Tc-MIBI MPI with other diagnostic methods, such as cardiac magnetic resonance imaging (CMRI), echocardiogram, and comprehensive electrocardiogram-related examinations (CEEs) to evaluate the diagnostic value of ^99m^Tc-MIBI MPI in detecting myocardial ischemia. In this study, we aim to introduce a novel perspective on the diagnosis of myocardial ischemia in pediatric patients, and to establish a clinically feasible and optimal non-invasive method for detecting myocardial ischemia in children with CALs after KD.

## Methods

### Study Population

**Fig. 1:**
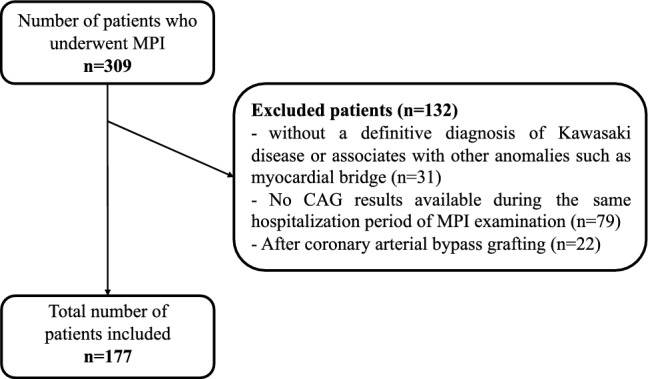
Study Flowchart. *MPI* ^99m^Tc-MIBI myocardial perfusion imaging, *CAG* coronary artery angiography

A total of 309 pediatric patients who underwent MPI at Children’s Hospital of Fudan University between July 2019 and February 2023 were included. 152 cases were excluded from the study due to the absence of a definitive diagnosis of KD or presence of other anomalies such as myocardial bridge, history of coronary artery revascularization, and lack of concurrent CAG during the same hospitalization. Finally, a total of 177 children were included for analysis in this study (Fig. 1). The study was approved by the Institutional Review Board of Children’s Hospital of Fudan University (Approval No. 2022-391), in accordance with the Declaration of Helsinki. All clinical data was de-identified.

The diagnosis of KD followed the 2017 American Heart Association (AHA) guidelines [1] and the Japanese diagnostic guideline (6th revised edition) [16]. Coronary artery aneurysm (CAA) inner diameter was measured by CAG, and the Z-score of coronary arteries was calculated by the online calculator (https://www.pedz.de/de/pedz/mmode.html). According to AHA guidelines, CAA was classified as small (2.5 ≤ Z-score < 5), medium (5 ≤ Z-score < 10), and giant (Z-score ≥ 10 or absolute inner diameter ≥ 8 mm) [17].

### Imaging Examination Methods and Diagnostic Criteria

#### CAG

All patients underwent CAG using the Siemens (AXIOM Artis dBA Detector System)/ Canon (INFX-9000 V) DSA machine through femoral punctures. Aortic root angiography and selective CAG were performed to visualize the left main coronary artery (LMCA), left anterior descending branch (LAD), left circumflex branch (LCX), and right coronary artery (RCA) from at least two projections. Significant stenosis of coronary artery on CAG was defined as either 50% luminal narrowing of the LMCA or 70% luminal narrowing of the remaining of coronary arteries [18]. The CAG results were analyzed and confirmed by at least two blinded pediatric cardiologists.

#### ^99m^Tc-MIBI MPI

^99m^Tc-MIBI MPI were performed by the dual-head Siemens E.CAM SPECT system. Patients would regularly undergo both adenosine triphosphate (ATP)-induced MPI and resting MPI. If a patient presents with evidence of significant myocardial ischemia such as history of myocardial infarction or decreased systolic function in echocardiogram, only a resting MPI will be performed.

The protocol of ATP-induced ^99m^Tc-MIBI MPI is as follows: (1) Prior to the test, intake of dipyridamole and theophylline was discontinued for 24 h, refrained from consuming food for 4–6 h, and beverages containing caffeine were avoided. (2) In a supine position, bilateral intravenous access was established and intravenous drip of ATP at a rate of 0.14 mg/kg/min (equal to 0.84 mg/kg) was initiated for 6 min. 3 min after the start of the drip, the contrast agent was injected into the contralateral vein. (3) Continuous monitoring of the electrocardiogram (ECG) was performed throughout the ATP infusion. Heart rate, blood pressure, ECG performance, and any symptoms at three time points were recorded: 3 min after the start of the drip, the end of the drip, and 5 min after completing the drip. (4) After the injection of contrast agent, high-fat foods such as cake or milk were consumed within 30 min, followed by MPI within 60 min. (5) Tomographic images of the left ventricular short axis, vertical axis, and horizontal long axis at a zoom level of 1.45, matrix size of 128*128, and 30 s per frame were obtained. (6) If the stress test yielded a positive result, resting MPI was performed 24 h later.

The presence of sparse or faulty distribution of the radiographic agent across two or more levels was considered indicative of myocardial ischemia [19]. If a radioactive defect was observed in the stress image but was filled in the rest image, it suggested reversible myocardial ischemia.

#### CMRI

CMRI was performed by the Siemens MR Avanto 1.5 T equipment (Germany). Examination techniques included Trufi sagittal, coronal, transverse, 2-chamber, 4-chamber, short-axis, short-axis T1WI, T2WI, T2WI SPAIR, myocardial perfusion, delayed enhancement, short-axis + 4-chamber cine sequence, thin 3D layer. The presence of delayed T2WI elevation, under-perfusion, or signal anomalies was considered indicative of myocardial ischemia in CMRI [20].

#### Echocardiogram

The presence of any of the following conditions was considered indicative of myocardial ischemia in echocardiogram: (1) decreased left ventricular ejection fraction (LVEF, < 50%) or fraction shortening (LVFS, < 25%); (2) decreased or absence of ventricular wall motion; (3) paradoxical motion or systole asynchrony [21] evaluated by at least two pediatric cardiologists. Different types of ventricular wall motion were defined as follows: (1) normal motion, characterized by a ventricular wall wave amplitude > 5 mm, (2) decreased motion, characterized by a ventricular wall wave amplitude 2–5 mm, and (3) loss of motion, characterized by a ventricular wall wave amplitude < 2 mm.

#### Comprehensive Electrocardiogram-Related Examinations

CEEs include 12-Lead electrocardiogram (ECG), 24-h Holter monitor (Holter), and exercise treadmill stress test (ETST). The presence of any of the following conditions was considered indicative of myocardial ischemia. (1) ECG: a 0.1 mV deviation in the resting ST segment from baseline and/or T-wave abnormalities (T-wave inversion or T-wave amplitude less than 1/10 of the R-wave in the same leads) [22, 23]. (2) Holter: horizontal or downward-sloping ST-segment depression 0.1mv for > 1 min [24, 25]. (3) ETST: ETST examination followed the modified Bruce-1 protocol for children. A positive ETST diagnosis was defined as the occurrence of the typical angina pectoris, ST-segment horizontal or downward-sloping depression 0.1 mV (60–80 ms after the J-point) lasting > 1 min, or severe arrhythmia during the test [26].

#### Statistical Analysis

Statistical analysis was performed using SPSS (version 26, IBM SPSS Statistics for Mac). Continuous data were presented as mean ± SD. The student’s *t*-test and Mann–Whitney *U* test were used for analysis. Categorical data were compared using the chi-squared test and presented as numbers (n) and percentages (%). Sensitivity, specificity, positive predictive value (PPV), and negative predictive value (NPV) of the diagnostic values of each examination were determined and displayed in a 2*2 table. Agreement between different tests was estimated using the Kappa statistic: Kappa < 0.40 was regarded as poor consistency, 0.40 ≤ Kappa < 0.75 was regarded as fair consistency, Kappa ≥ 0.75 was regarded as good consistency. Statistics were considered as significant when *P* < 0.05.

## Results

### Baseline Characteristics of Patients

There were 142 (80.2%) males and 35 (19.8%) females in the final analysis dataset. The mean age was 5.75 ± 3.77 years (range: 6 months to 16 years); the mean duration after KD onset was 2.75 ± 2.99 years (range: 2 months to 14 years). CAG, echocardiogram, ECG, and MPI were performed in all patients, and CMRI was performed in 121 patients. (Table 1). Table 2 describes the characteristics of CAL in CAG. There were 48 (27.1%) patients who had significant coronary artery stenosis (LMCA: 4 patients, ≥ 70%; the remaining coronary artery: 44 patients, ≥ 70%), and 111 (62.7%) patients had at least one GCAA in all coronary arteries (Table 2).

**Table 1:** Baseline characteristics (*n* = 177)

Variable	
Male, *n* (%)	142 (80.2%)
Age (years) (mean ± SD)	5.75 ± 3.77
Height (cm) (mean ± SD)	111.69 ± 24.19
Weight (kg) (mean ± SD)	23.49 ± 13.62
Duration after KD onset (years) (mean ± SD)	2.75 ± 2.99
Days between MPI and CAG (mean ± SD)	6.32 ± 11.81
Positive results of each test* [positive no./no. who received the test (positive ratio)]	
MPI, *n* (%)	78/177 (44.1%)
CMRI, *n* (%)	32/121 (26.4%)
Echocardiogram, *n* (%)	16/177 (9.0%)
CEEs, *n* (%)	43/177 (24.3%)
ECG, *n* (%)	20/177 (11.3%)
Holter, *n* (%)	28/142 (19.7%)
ETST, *n* (%)	9/74 (12.2%)

*MPI* ^99m^Tc-MIBI myocardial perfusion imaging, *CAG* coronary artery angiography, *CMRI* cardiac magnetic resonance imaging, *CEEs* comprehensive electrocardiogram-related examinations, *ECG* 12-Lead electrocardiogram, *ETST* exercise treadmill stress test

*For the diagnosis of myocardial ischemia

**Table 2:** Characteristics of CAL in CAG (*n* = 177)

Variable	
Significant coronary artery stenosis, *n* (%)	
No	129 (72.9%)
Yes	48 (27.1%)
CAA, *n* (%)	
No	15 (8.5%)
Small CAA	13 (7.3%)
Medium CAA	%1 38 (21.5%)
Giant CAA	111 (62.7%)
The location of CAL, *n* (%)	
LMCA	99 (55.9%)
LAD	135 (76.3%)
LCX	64 (36.2%)
RCA	150 (84.8%)
The number of arteries involved, *n* (%)	
One	21 (11.9%)
Two	61 (34.5%)
Three and above	94 (53.1%)

*CAL* coronary artery lesion, *CAG* coronary artery angiography, *CAA* coronary artery aneurysm, *LMCA* left main coronary artery, *LAD* left anterior descending branch, *LCX* left circumflex branch, *RCA* right coronary artery

### Safety Evaluation of ATP-Induced ^99m^Tc-MIBI MPI

Among 177 patients who underwent ^99m^Tc-MIBI MPI, 28 (19.1%) patients only received resting MPI, 149 (84.2%) patients (age range: 7 months to 16 years) received ATP-induced stress MPI, and 40 (26.9%) patients experienced mild discomfort during the MPI procedure, which was resolved spontaneously. These included chest tightness (16/40), sweating (11/40), nausea (9/40), palpitations (8/40), shortness of breath (7/40), skin flushing (4/40), and dizziness (4/40).

### Diagnostic Performance of MPI and Other Non-invasive Examinations

Combining with the presence or absence of significant coronary artery stenosis in CAG, the results of each examination for myocardial ischemia are demonstrated in Table 3. MPI had the highest positive rate among all non-invasive examinations for detecting myocardial ischemia, which was 44.1% (78/177). The positive rate of CMRI was 25.4% (32/121), echocardiogram was 9.0% (16/177), and CEEs was 24.3% (43/177).

**Table 3:** Diagnostic results of each test for myocardial ischemia

Diagnostic test	Myocardial ischemia	Significant coronary artery stenosis*		Total
		Yes	No	
MPI	Yes	38 (21.5%)	40 (22.6%)	78 (44.1%)
	No	10 (5.6%)	89 (50.3%)	99 (55.9%)
Total		48 (27.1%)	129 (72.9%)	177
CMRI	Yes	23 (19.0%)	9 (7.4%)	32 (26.4%)
	No	11 (9.1%)	78 (64.5%)	89 (73.6%)
Total		34 (28.1%)	87 (71.9%)	121
Echocardiogram	Yes	12 (6.8%)	4 (2.25%)	16 (9.0%)
	No	36 (20.3%)	125 (70.6%)	161 (90.9%)
Total		48 (27.1%)	128 (72.3%)	177
CEEs	Yes	25 (14.1%)	18 (10.2%)	43 (24.3%)
	No	23 (13.0%)	111 (62.7%)	134 (75.7%)
Total		48 (27.1%)	128 (72.3%)	177

*MPI* ^99m^Tc-MIBI myocardial perfusion imaging, *CAG* coronary artery angiography, *CMRI* cardiac magnetic resonance imaging, *CEEs* comprehensive electrocardiogram-related examinations

*The definition of significant coronary artery stenosis was either 50% luminal narrowing of the LMCA or 70% luminal narrowing of the remaining of coronary arteries based on the CAG examination [18]

The diagnostic values of each non-invasive examination for myocardial ischemia are illustrated in Table 4. The sensitivity of MPI was 79.2%, which was superior to CMRI (67.7%; *P* = 0.037) and echocardiogram (25.0%; *P* = 0.04). The negative predictive value (NPV) of MPI was 89.9%, which was comparable to CMRI (87.6%; *P* = 0.108) and CEEs (82.8%;* P* = 0.760) but superior to echocardiogram (77.6%; *P* = 0.004).

**Table 4:** Diagnostic value in detecting myocardial ischemia of MPI and other examinations

Test	Sensitivity	Specificity	Accuracy	PPV	NPV	Kappa
MPI	79.2%	69.0%	71.8%	48.7%	89.9%	0.403
	(38/48)	(89/129)	(127/177)	(38/78)	(89/99)	
CMRI	67.7%*	89.7%	83.5%*	71.9%	87.6%	0.583
	(23/34)	(78/87)	(101/121)	(23/32)	(78/89)	
Echocardiogram	25.0%*	96.9%	77.4%	75.0%	77.6%**	0.277
	(12/48)	(12/129)	(137/177)	(12/16)	(125/160)	
CEEs	52.1%	85.9%	76.8%	58.1%	82.7%	0.394
	(25/48)	(110/128)	(136/177)	(25/43)	(111/134)	

*MPI* ^99m^Tc-MIBI myocardial perfusion imaging, *CMRI* cardiac magnetic resonance imaging, *CEEs* comprehensive electrocardiogram-related examinations, *PPV* positive predictive value, *NPV* negative predictive value

Compared to MPI: *P < 0.05; **P < 0.01

In contrast, the specificity of MPI was 69.0%, showing no statistical difference from other examinations (*P* > 0.05) and the accuracy of MPI was 71.8%, which was lower than the CMRI (83.5%, *P* = 0.039). Regarding diagnostic consistency (Kappa) with CAG, MPI, CMRI, and CEEs demonstrated moderate consistency (0.4–0.75), all of which performed better than echocardiogram (Table 4).

**Fig. 2:**
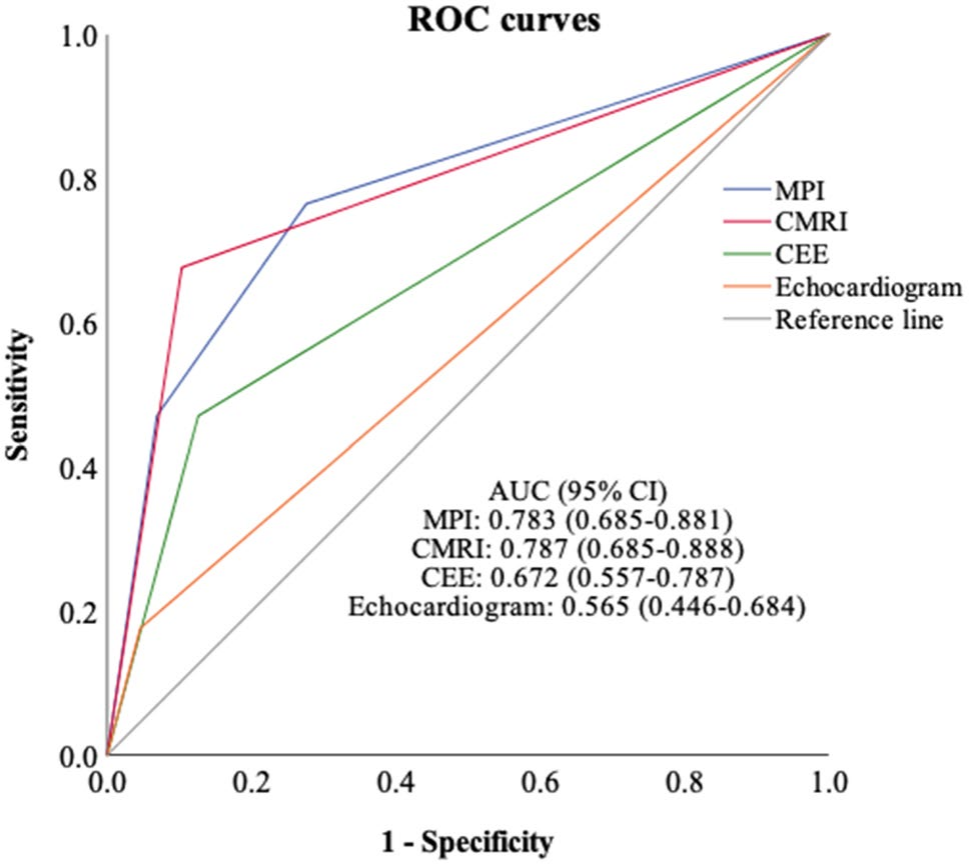
Receiver-operating characteristic curves (ROC) with corresponding area under the curves (AUC). *MPI* ^99m^Tc-MIBI myocardial perfusion imaging, *CAG* coronary artery angiography, *CMRI* cardiac magnetic resonance imaging, *Echo* echocardiogram, *CEEs* comprehensive electrocardiogram-related examinations

In addition, to display the receiver-operating characteristic (ROC) curve (Fig. 2), we quantified the myocardial ischemia results of MPI into three grades: no myocardial ischemia (0 point), reversible myocardial ischemia (1 point), and irreversible myocardial ischemia (2 points). The area under the curve (AUC) of the MPI examinations was discovered to be 0.783, which was higher than the AUC of echocardiogram (0.565) and CEEs (0.672) but lower than that of CMRI (0.787).

### Diagnostic Value of MPI Combined with CMRI in Detecting Myocardial Ischemia

Considering the diagnostic sensitivity advantage of MPI and the accuracy advantage of CMRI for detecting myocardial ischemia in pediatric patients with CAL after KD, we developed the “Tandem” and “Parallel” combined diagnostic methods of MPI and CMRI (Table 5). In the “Parallel” strategy, a positive result from either MPI or CMRI would be considered indicative of myocardial ischemia. In the “Tandem” strategy, positive results from both MPI and CMRI would be considered indicative of myocardial ischemia.

**Table 5:** Diagnostic results of MPI combined with CMRI

Diagnostic test	Myocardial ischemia	Significant coronary artery stenosis		Total
		Yes	No	
“Tandem” MPI + CMRI	Yes	20 (16%)	4 (3.2%)	24 (16.1%)
	No	16 (12.8%)	109 (87.2%)	125 (83.9%)
Total		36 (24.2%)	113 (75.8%)	149
“Parallel” MPI/CMRI	Yes	41 (27.5%)	45 (30.2%)	86 (57.7%))
	No	5 (3.4%)	58 (38.9%)	63 (42.3%)
Total		46 (30.9%)	113 (75.8%))	149

As a result (Table 6), the “Tandem” strategy improved the diagnostic specificity (96.5%, 109/113), accuracy (86.6%, 129/149), PPV (83.3%, 20/24), and consistency (Kappa = 0.587) (*P* < 0.001). The “Parallel” strategy enhanced the diagnostic sensitivity (89.1%, 41/46) and NPV (92.1%, 58/63) (*P* < 0.001).

**Table 6:** Diagnostic value of MPI, CMRI, “Tandem,” and “Parallel” for myocardial ischemia

Test	Sensitivity	Specificity	Accuracy	PPV	NPV	Kappa
MPI	79.2%	69.0%	71.8%	48.7%	89.9%	0.403
	(38/48)	(89/129)	(127/177)	(39/78)	(89/99)	
CMRI	67.7%	89.7%	83.5%	71.9%	87.6%	0.583
	(23/34)	(78/87)	(101/121)	(23/32)	(78/89)	
“Tandem” MPI + CMRI	55.6%	96.5%	86.6%	83.3%	87.2%	0.587
	(20/36)	(109/113)	(129/149)	(20/24)	(109/125)	
“Parallel” MPI/CMRI	89.1%	56.3%	66.4%	47.7%	92.1%	0.366
	(41/46)	(58/103)	(99/149)	(45/86)	(58/63)	

Additionally, 4 (3.2%) cases were diagnosed with myocardial ischemia using the “Tandem” strategy despite the absence of significant coronary artery stenosis in CAG, which was considered as “false-positive” (FP). 5 (3.4%) cases were not diagnosed with myocardial ischemia using the “Parallel” strategy, despite significant coronary artery stenosis being observed in CAG of these patients. They were considered as “false-negative” (FN).

## Discussion

Pediatric patients with CAL after KD are prone to myocardial ischemia and even infarction. In the long-term follow-up study of 146 KD patients with CAA (follow-up period: 10–21 years), the incidence of myocardial infarction was 1.9% (11/146) with a mortality rate of 0.8% (5/594) [27]. Among patients who developed myocardial infarction, 8 (73%) patients had GCAA and 3 (27.3%) patients had medium CAA, and 5 (45.4%) patients had asymptomatic myocardial infarction. Therefore, to establish a clinically feasible and accurate method, periodical myocardial monitoring is crucial for improving the prognosis of pediatric patients with CAL after KD.

Due to the invasiveness, radioactivity, complexity, and higher risk of CAG and FFR, particularly in infants, which represents a higher risk population for more severe coronary artery involvement, they are not suited for periodical monitoring in pediatric patients with CAL after KD [28]. Previous studies in adults and children have assessed the diagnostic performance of non-invasive examinations in myocardial ischemia evaluation including inducible myocardial ischemia. ECG (including Holter) and echocardiogram are simple to perform and easy to repeat, so these are considered as the primary examinations and widely used in clinical practice. However, the sensitivity and specificity of ECG and echocardiogram in detecting myocardial ischemia are low [29]. ETST can only applied in children older than 6 years old, and the specificity, sensitivity, and accuracy are not high enough, with only about 77%, 68%, and 50% [30, 31].

Since 1997, ^99m^Tc-MIBI MPI has been used in pediatric patients with CAL after KD [8, 32]. Beamish et al. [33] reported a case of a 14-year-old male with a history of KD, who exhibited no clinical symptoms of myocardial ischemia and presented with negative ETST results. MPI revealed notable defects under stress conditions using intravenous persantine (dipyridamole), which became normal at resting MPI. Studies in pediatric patients with hypertrophic cardiomyopathy have supported the clinical benefit of stressed MPI in detecting inducible myocardial ischemia [34].

In this study, we retrospectively collected a large cohort of pediatric patient with CALs after KD from 6 months to 16 years old. Study results recognized the safety and feasibility of ATP-stressed ^99m^Tc-MIBI MPI examination, as well as its diagnostic value in detecting inducible myocardial ischemia in these pediatric patients. Our data showed that MPI had the highest sensitivity (79.17%) among non-invasive examinations, but the accuracy and consistency of MPI were slightly inferior to CMRI. The AUC of MPI was significantly superior to those of echocardiogram and CEES (*P* < 0.05). We also noticed that the “Tandem” strategy (MPI + CMRI) can significantly improve the specificity, accuracy, PPV, and consistency in the diagnosis of myocardial ischemia with the reference of CAG results. Therefore, whenever feasible, both MPI and CMRI should be included in the regular follow-up protocol for pediatric patients with CAL after KD.

**Fig. 3:**
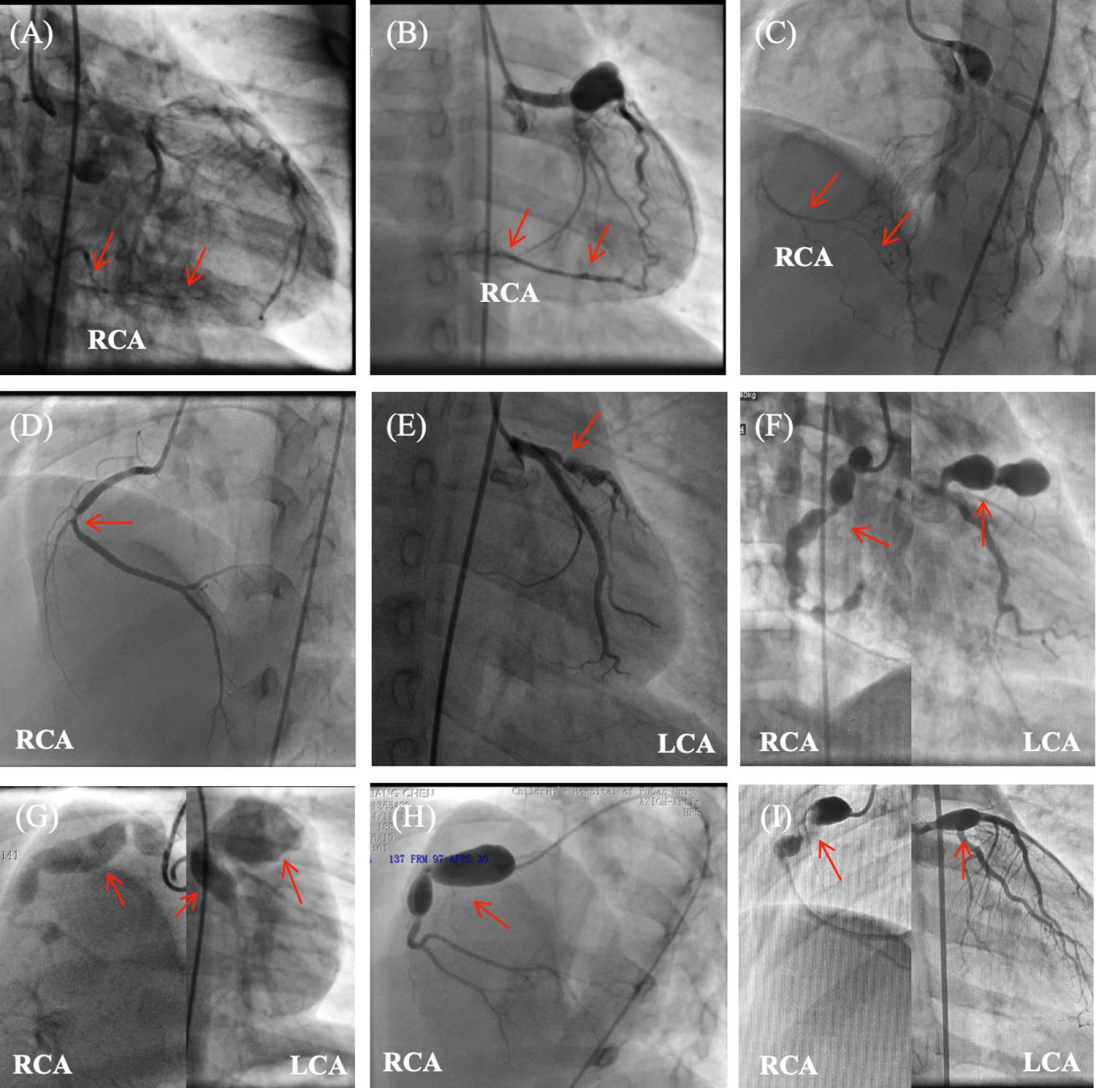
Angiograms of coronary artery lesions in special cases. **A**–**E** Angiograms of 5 false-positive patients including widespread collateral arteries to occluded distal RCA in 3 patients (**A**–**C**, arrows) and significant stenosis of RCA (**D**, arrow) and LAD (**E**, arrow). **F**–**I** Angiograms of 4 false-negative patients showed complex and multiple huge aneurysms (arrows) in both LCA and RCA. *LCA* left coronary artery, *RCA* right coronary artery, *LAD* left anterior descending branch

However, we found that the diagnosis of myocardial ischemia in some specific cases using the composite strategies conflicted with the results of CAG. To identify the reasons, we further analyzed the characteristics of coronary artery in CAG of these cases. Among 5 FN cases, 3 patients developed ideal collateral circulations, which were able to provide enough blood supply for myocardium despite the occlusion of right coronary artery (Fig. 3A–C). In the remaining 2 patients, one had RCA stenosis (70%, Fig. 3D) and the other has LAD stenosis (90%, Fig. 3E). It suggested that non-invasive myocardial ischemia assessment examinations may not achieve sufficient diagnostic sensitivity. Quantitative, invasive examinations such as FFR are necessary when making decisions on coronary artery revascularization. On the other hand, multiple “sausages-like” or “bead-like” GCAAs were observed in all 4 FP cases exhibited. The complex and abnormal anatomical structure of multiple GCAAs could increase the likelihood of endothelial dysfunction and hemodynamic abnormalities, such as “uneven” and “extremely slow” blood flow within GCAA. Consequently, delayed visualization of distal end of coronary artery after several cardiac cycles may occur (Fig. 3F–I). This suggested that myocardial ischemia may exist in cases of multiple GCAAs without significant coronary artery stenosis, and the judgment of FP may be incorrect.

Our study has several limitations. Firstly, this was a retrospective study. Secondly, because patients did not receive all the non-invasive examinations during follow-up, we included patients who had undergone at least both ^99m^Tc-MIBI MPI and CAG for analysis. Among these patients, approximately 68% (121/177) had undergone CMRI, providing a sufficient amount of data for our analysis. Finally, due to the inconsistence of the baseline characteristics of cohort, it may raise the possibility of statistical bias. To address this issue, a randomized controlled trail (RCT) will be conducted to ensure that every participant receives the series of non-invasive examinations, including ^99m^Tc-MIBI MPI, and CMRI.

This is the first and largest retrospective study to systematically compare the diagnostic value of different diagnostic examinations for myocardial ischemia assessment in pediatric patients with CAL after KD. Combining with the results of CAG, we recognized the safety, feasibility, and diagnostic values of ^99m^Tc-MIBI MPI. MPI has optimal diagnostic sensitivity among non-invasive examinations, including CMRI, echocardiogram, and CEE. Moreover, the composite monitoring strategies of MPI and CMRI can effectively improve the accuracy of myocardial ischemia diagnosis. Notably, FP cases especially with multiple GCAAs should be taken seriously as they may increase the risk of myocardial ischemia even in the absence of significant stenosis. Further studies are needed to confirm this finding, which can provide clinical evidence for long-term management and treatment, such as coronary artery revascularization, in pediatric patients with GCAAs after KD.

## References

[1] Mccrindle BW, Rowley AH, Newburger JW et al (2017) Diagnosis, treatment, and long-term management of Kawasaki disease: a scientific statement for health professionals from the American heart association. Circulation 135(17):e927–e999 28356445 10.1161/CIR.0000000000000484

[2] Seki M, MinamI T (2022) Kawasaki disease: pathology, risks, and management. Vasc Health Risk Manag 18:407–416 35711626 10.2147/VHRM.S291762 PMC9196282

[3] Xie LP, Yan WL, Huang M et al (2020) Epidemiologic features of Kawasaki disease in Shanghai from 2013 through 2017. J Epidemiol 30(10):429–435 31548437 10.2188/jea.JE20190065 PMC7492704

[4] De Graeff N, Groot N, Ozen S et al (2019) European consensus-based recommendations for the diagnosis and treatment of Kawasaki disease—the SHARE initiative. Rheumatology (Oxford) 58(4):672–682 30535127 10.1093/rheumatology/key344

[5] Mu ZL, Jiao FY, Xie KS (2021) Interpretation of the JCS/JSCS 2020 guideline on diagnosis and management of cardiovascular sequelae in Kawasaki disease. Zhongguo Dang Dai Er Ke Za Zhi 23(3):213–220 33691912 10.7499/j.issn.1008-8830.2010134 PMC7969191

[6] Weustink AC, Mollet NR, Neefjes LA et al (2010) Diagnostic accuracy and clinical utility of noninvasive testing for coronary artery disease. Ann Intern Med 152(10):630–639 20479028 10.7326/0003-4819-152-10-201005180-00003

[7] Uren NG, Melin JA, De Bruyne B et al (1994) Relation between myocardial blood flow and the severity of coronary-artery stenosis. N Engl J Med 330(25):1782–1788 8190154 10.1056/NEJM199406233302503

[8] Miyagawa M, Mochizuki T, Murase K et al (1998) Prognostic value of dipyridamole-thallium myocardial scintigraphy in patients with Kawasaki disease. Circulation 98(10):990–996 9737519 10.1161/01.cir.98.10.990

[9] Ora M, Gambhir S (2019) Myocardial perfusion imaging: a brief review of nuclear and nonnuclear techniques and comparative evaluation of recent advances. Indian J Nucl Med 34(4):263–270 31579355 10.4103/ijnm.IJNM_90_19 PMC6771197

[10] Perrone MA, Cimini A, Ricci M et al (2023) Myocardial functional imaging in pediatric nuclear cardiology. J Cardiovasc Dev Dis 10(9):361 37754790 10.3390/jcdd10090361 PMC10531976

[11] Danad I, Szymonifka J, Twisk JWR et al (2017) Diagnostic performance of cardiac imaging methods to diagnose ischaemia-causing coronary artery disease when directly compared with fractional flow reserve as a reference standard: a meta-analysis. Eur Heart J 38(13):991–998 27141095 10.1093/eurheartj/ehw095 PMC5381594

[12] Takx RA, Blomberg BA, El Aidi H et al (2015) Diagnostic accuracy of stress myocardial perfusion imaging compared to invasive coronary angiography with fractional flow reserve meta-analysis. Circ Cardiovasc Imaging. 10.1161/CIRCIMAGING.114.002666 25596143 10.1161/CIRCIMAGING.114.002666

[13] Underwood SR, Anagnostopoulos C, Cerqueira M et al (2004) Myocardial perfusion scintigraphy: the evidence. Eur J Nucl Med Mol Imaging 31(2):261–291 15129710 10.1007/s00259-003-1344-5 PMC2562441

[14] Srinivasan R, Weller R, Chelliah A et al (2016) Multimodality cardiac imaging in a patient with Kawasaki disease and giant aneurysms. Case Rep Pediatr 2016:4298098 27872783 10.1155/2016/4298098 PMC5107831

[15] Sato T, Ushiroda Y, Oyama T et al (2012) Kawasaki disease-associated MERS: pathological insights from SPECT findings. Brain Dev 34(7):605–608 22019463 10.1016/j.braindev.2011.09.015

[16] Kobayashi T, Ayusawa M, Suzuki H et al (2020) Revision of diagnostic guidelines for Kawasaki disease. Pediatr Int 62(10):1135–8 33001522 10.1111/ped.14326

[17] Nakazato R, Berman DS, Alexanderson E et al (2013) Myocardial perfusion imaging with PET. Imaging Med 5(1):35–46 23671459 10.2217/iim.13.1 PMC3650901

[18] Laspas F, Pipikos T, Karatzis E et al (2020) Cardiac magnetic resonance versus single-photon emission computed tomography for detecting coronary artery disease and myocardial ischemia: comparison with coronary angiography. Diagnostics (Basel) 10(4):190 32235380 10.3390/diagnostics10040190 PMC7235742

[19] Van Diemen PA, Driessen RS, Kooistra RA et al (2020) Comparison between the performance of quantitative flow ratio and perfusion imaging for diagnosing myocardial ischemia. JACC Cardiovasc Imaging 13(9):1976–1985 32305469 10.1016/j.jcmg.2020.02.012

[20] Sirajuddin A, Mirmomen SM, Kligerman SJ et al (2021) Ischemic heart disease: noninvasive imaging techniques and findings. Radiographics 41(4):990–1021 34019437 10.1148/rg.2021200125 PMC8262179

[21] Breithardt OA, Stellbrink C, Kramer AP et al (2002) Echocardiographic quantification of left ventricular asynchrony predicts an acute hemodynamic benefit of cardiac resynchronization therapy. J Am Coll Cardiol 40(3):536–545 12142123 10.1016/s0735-1097(02)01987-3

[22] Rautaharju PM, Surawicz B, Gettes LS et al (2009) AHA/ACCF/HRS recommendations for the standardization and interpretation of the electrocardiogram: part IV: the ST segment, T and U waves, and the QT interval: a scientific statement from the American heart association electrocardiography and arrhythmias committee, council on clinical cardiology; the American college of cardiology foundation; and the heart rhythm society. Endorsed by the international society for computerized electrocardiology. J Am Coll Cardiol 53(11):982–91 19281931 10.1016/j.jacc.2008.12.014

[23] Ed Burns M C. Myocardial Ischaemia. https://litfl.com/myocardial-ischaemia-ecg-library/; home ECG Library. 2022

[24] Fletcher GF, Balady G, Froelicher VF et al (1995) Exercise standards: a statement for healthcare professionals from the American heart association. Circulation 91(2):580–615 7805272 10.1161/01.cir.91.2.580

[25] Delfino RJ, Gillen DL, Tjoa T et al (2011) Electrocardiographic ST-segment depression and exposure to traffic-related aerosols in elderly subjects with coronary artery disease. Environ Health Perspect 119(2):196–202 20965803 10.1289/ehp.1002372 PMC3040606

[26] Liu Fang ZQ (2021) Assessment for myocardial ischemia related to coronary artery abnormalities in children with Kawasaki disease. Chin J Pract Pediatr 36(05):336–339

[27] Kato H, Sugimura T, Akagi T et al (1996) Long-term consequences of Kawasaki disease: a 10-to 21-year follow-up study of 594 patients. Circulation 94(6):1379–1385 8822996 10.1161/01.cir.94.6.1379

[28] Gaede L, Moellmann H, Rudolph T et al (2019) Coronary angiography with pressure wire and fractional flow reserve: state of the art in the diagnosis of coronary stenosis. Dtsch Arztebl Int 116(12):205 31056086 10.3238/arztebl.2019.0205 PMC6514382

[29] Jone PN, Romanowicz J, Browne L et al (2022) Imaging evaluation of Kawasaki disease. Curr Cardiol Rep 24(10):1487–1494 35986822 10.1007/s11886-022-01768-4

[30] Pais P (2018) Treadmill stress tests should not be part of “routine health check package.” Indian Heart J 70:934–936 30580868 10.1016/j.ihj.2018.09.010 PMC6306354

[31] Lau GT, Wei H, Wickham J et al (2018) The significance of equivocal exercise treadmill ECG for intermediate risk chest pain assessment—insight from coronary CT angiography data. Heart Lung Circ 27(1):50–57 28320636 10.1016/j.hlc.2017.01.015

[32] Karasawa K, Ayusawa M, Noto N et al (1997) Optimum protocol of technetium-99m tetrofosmin myocardial perfusion imaging for the detection of coronary stenosis lesions in Kawasaki disease. J Cardiol 30(6):331–339 9436075

[33] Beamish J, O’Connell MJ, El Khuffash A et al (2006) Calcified occlusion of the right coronary artery in Kawasaki disease: evidence of myocardial ischaemia using cardiac technetium-99m-tetrofosmin perfusion single-photon emission computed tomography. Arch Dis Child 91(11):926–928 17056866 10.1136/adc.2006.099630 PMC2082938

[34] Ziolkowska L, Boruc A, Sobielarska-Lysiak D et al (2021) Prognostic significance of myocardial ischemia detected by single-photon emission computed tomography in children with hypertrophic cardiomyopathy. Pediatr Cardiol 42(4):960–968 33687492 10.1007/s00246-021-02570-9 PMC8110494

